# Peripartal pain perception and pain therapy: introduction and validation of a questionnaire as a quality instrument

**DOI:** 10.1007/s00404-021-06246-w

**Published:** 2021-09-20

**Authors:** A. Linzbach, D. Nitschke, J. Rothaug, M. Komann, C. Weinmann, E. Schleußner, W. Meißner, J. Jimenez Cruz, U. Schneider

**Affiliations:** 1 Department of Obstetrics, Division of Prenatal Diagnostics and Fetal Physiology, University Hospital Jena, Am Klinikum 1, 07747 Jena, Germany; 2 Department of Anesthesiology and Intensive Care Medicine, Section Pain Therapy, University Hospital Jena, Jena, Germany; 3 Department of Obstetrics and Prenatal Medicine, University Hospital Bonn, Bonn, Germany

**Keywords:** Labor pain, Questionnaire, Pain measurement, Pain therapy, Vaginal birth, NRS—numeric rating scale

## Abstract

**Background:**

Labor pain is difficult to measure. The aim of this proof-of-concept study is to implement and test a questionnaire assessing pain sensation during and after vaginal deliveries. Its key aspect is a highly standardized survey of patient-reported outcome (PRO) by staff not involved in routine care.

**Methods:**

Between January and November 2015 339 women were assessed 24–48 h after spontaneous or operative-vaginal delivery of a singleton. German language skills were a prerequisite to participate. The test–retest reliability was calculated in 38 women 24–36 and 48–72 h postpartum between July and October 2017. Primiparae after spontaneous delivery and multiparae with no history of operative deliveries were compared in a subgroup analysis.

**Results:**

Maximum labor pain and post-partum pain were reported a median of 9 [8–10] and 4 [3–6]. Higher ratings were associated with younger age, higher gestational ages, infant’s biometrics, and the duration of laboring.

Only regional analgesia tended to reduce pain perception (NRS 8 vs. 9). Higher-degree injuries were associated with less pain postpartum. The questionnaire proved to be reliable in most aspects (Cronbach's α > 0.6 for 19/21 questions) and showed an acceptable content and criterion validity (Cohen correlation >  ± 0.3, interrelation between items).

**Conclusion:**

Labor is a very painful experience, irrespective of previous obstetric history. Ratings indicate inadequateness of treatment except for patients receiving preventive postoperative pain management. Systematic postpartum pain assessment, hence, is still a pending issue. Adjustments will be made concerning language skills and specific questions on effectiveness of analgesia otherwise good reliability and validity of the questionnaire were proven.

**Supplementary Information:**

The online version contains supplementary material available at 10.1007/s00404-021-06246-w.

## Introduction

Labor and giving birth are physiological processes, but also fundamentally painful experiences. Pain experience during childbirth depends on the interaction of multiple physiologic and psychosocial factors and should be assessed multi-dimensionally. It incorporates understanding the origin of labor pain stimuli and the mechanisms of pain modulation, but also the consideration of individual psychological aspects including suffering and respectability to consolation [1].

The major pain causing mechanisms during labor, especially during the progression of the presenting part of the fetus through the birth canal, are ischaemia of the pelvic organs and stretching of the pelvic floor [2, 3]. Cervical, myometrial, and peritoneal nociceptors in the uterus are stimulated during stage I of labor when the lower uterine segment and the cervix are stretched. Low velocity C-fibres mediate visceral, throbbing, colic-like pain to the segments Th10-S5 of the spinal cord, accompanied by vegetative phenomena. In the second stage, the stretching perineal fascia, perineal skin, and subcutaneous tissue lead to the perception of sharp somatic, well localized pain via S2-4.

Labor pain is individual and subject to a variety of complex influences based on personal expectations, previous experiences (obstetric and non-obstetric) and foreseeable and non-foreseeable obstetric aspects. Nowadays, expecting women tend to link the prospect of giving birth naturally with a desire for maximum medical safety. Medical staff on the other hand may underestimate the relevance of perinatal pain management by prejudging the physiological nature of the process [4, 5].

Several concepts are available for pain management during labor. Water baths, acupuncture or massages tend to positively influence the birth experience [6]. Close care during childbirth has proven to be particularly important. This not only leads to a reduction in surgical interventions, but also to a reduced need for pain relievers [7]. Pharmacological interventions range from non-opioids with spasmolytic effect (i.e., butylscopolamine via different routes of administration, sometimes in combination with paracetamol), inhalation agents (nitric oxide), opioids (meptazinol, pethidine, fentanyl, remifentanil, alfentanil) to regional analgesia. An epidural analgesia (synonymous peridural analgesia, PDA) is considered the gold-standard to relieve labor pain [8]. A PDA has proven most effective in reducing labor pain in randomized controlled trials [9, 10]. However, a PDA is an invasive procedure, requiring skilled staff and continuous monitoring, and is associated with rare but potentially severe risks. In addition, the use of an epidural analgesia should be incorporated into a multidimensional management. Although, epidural analgesia was previously thought to cause prolongation of labor, a higher rate of malpresentation and obstruction of labor, more recent studies could demonstrate that it did not increase the rates of operative-vaginal deliveries [10]. Taking into account the modern care strategies for pain control under labor, it seems that both appropriate choice of analgesic tools, depending on stage and progression of labor, and participation of the women in the decision-making process are decisive in the experience of pain [11].

Therefore, pain is difficult to measure. Improvement of labor-pain management is hardly achievable by conducting prospective, randomized controlled trials. Supplementary tools are required to adjust interventions to the needs of the individual based on robust epidemiological data and semi-quantitative measures.

The QUIPS project (Quality-Improvement-in-Postoperative-Pain-Management) is an initiative conducted by the Jena University Hospital and has been implemented for many years for internal and cross-clinic quality management of pain therapy based on the collection of patient-reported outcomes (PRO) and processed parameters in the context of surgical interventions. In the obstetric context, QUIPS was used in patients after cesarean section [12], demonstrating inadequate pain management in the majority of women. One key aspect of QUIPS is a highly standardized assessment of PRO by staff not involved in routine care, thus aiming to reduce questioning bias.

The objective of this study is to implement a questionnaire for the PRO assessment of peripartum pain sensation during and after vaginal, both spontaneous and operative-vaginal, deliveries. The questionnaire that is based on the established QUIPS template, is used in a monocentric proof-of-concept study to test its applicability, reliability, and validity. The results aim to uncover deficits in the current quality standards of the peripartum pain management in our department. Plausibility of the results is verified according to anticipated interrelations between solid obstetric data and the results of the survey.

## Methods

This study is a prospective, non-randomized, unblinded survey study on subjective pain experience during and after spontaneous or operative-vaginal delivery (outcome questionnaire) and its association with demographic and obstetric aspects (process questionnaire). The questionnaire were modified based on the original QUIPS template and will be integrated into the QUIPS project after adjustment. The local ethics committee of Jena University Hospital has approved the study.

The observation period covered the months from January to November 2015 at the Department of Obstetrics at Jena University Hospital. Staff not involved in peripartum care visited the maternity ward on 3–4 days per week. For Re-Test validation, a second survey period was performed from July 2017 to October 2017 (see Statistics section for details).

Eligible women were visited 24–48 h after vaginal delivery of a singleton, consented, and the questionnaire was handed to them and re-collected after 30 min. Since this study was conceived as a validation study of a questionnaire in German language, lack of German language skills had to be treated as an exclusion criterion.

The event progression and pain therapy data were collected from the birth register and the patient files of mother and newborn in the maternity ward. In addition to demographic data, these data provide details on the course of labor, delivery, birth injury, and the pain therapy administered during and after birth.

**Fig. 1:**
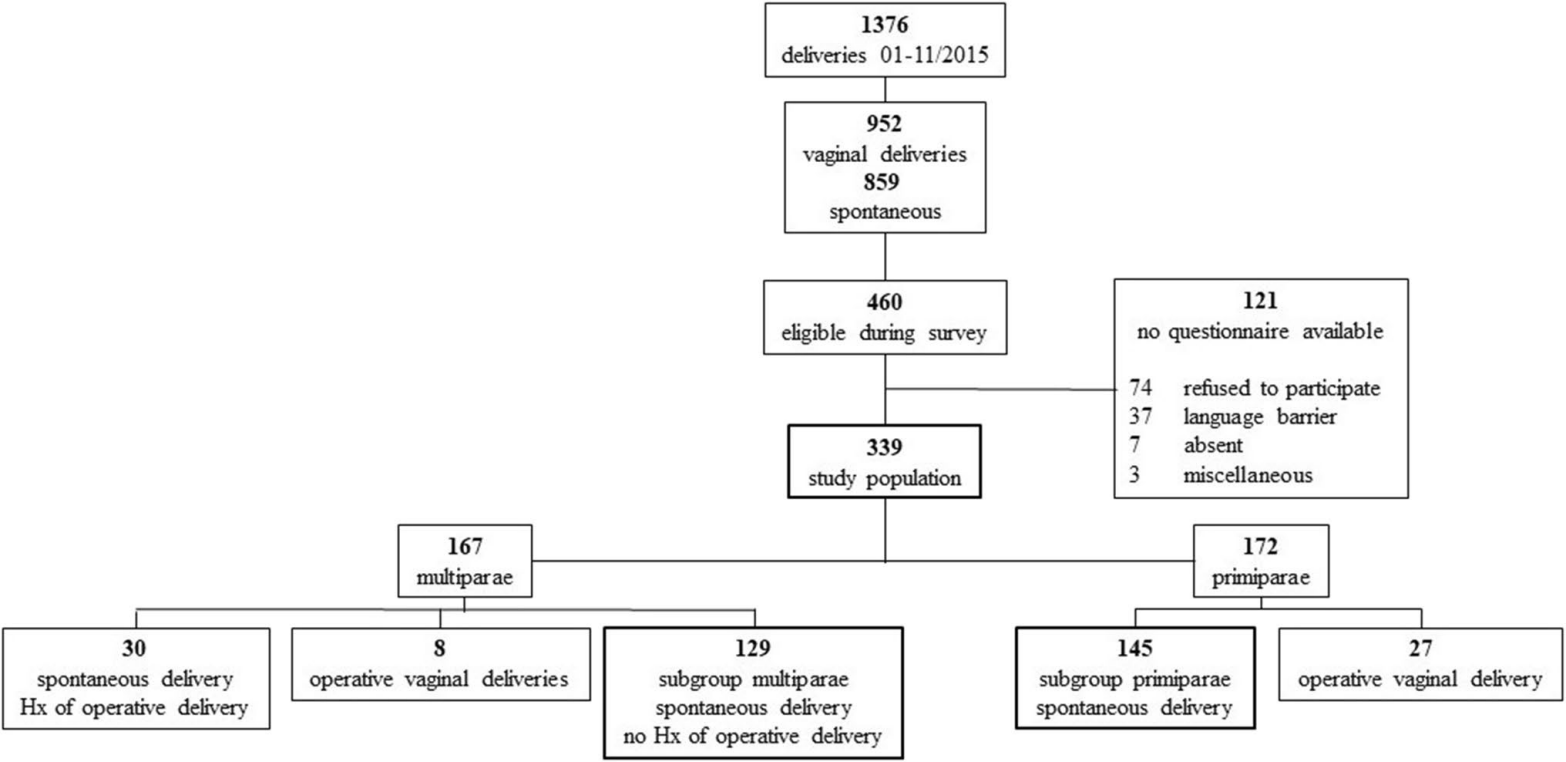
Flowchart of the study collective. Hx = history (anamnesis)

With the aim to differentiate co-variates and to check the plausibility of the results the subgroups of primiparae after spontaneous delivery [primiparae] and multiparae after spontaneous delivery with no history of operative deliveries [multiparae] were compared in a subgroup analysis (see Fig. 1 for details). The following co-variates were studied: maternal age, gestational age at birth, duration of birth, body metrics of the newborn, induction of labor, and method of induction.

The (outcome) questionnaire consists of three parts. The first part contains questions about pain and pain therapy during childbirth (questions 1–6). The second part focuses on pain and pain-related impairments, as well as pain therapy after childbirth (questions 7–13). The last section focuses on the general attitude towards pain therapy, perceived quality of care, as well as non-medicinal methods for pain relief (questions 14–19). The women were also asked to assess the extent and satisfaction with midwifery care during childbirth (questions 20 and 21). Pain intensity and satisfaction with pain therapy, as well as the care provided by the hospital staff were assessed using a numerical rating scale (NRS). Satisfaction and pain intensity was assessed using a 11-step numerical rating scale (NRS) with 0—‘very dissatisfied’ to 10—‘very satisfied’ and 0—‘no pain’ to 10—‘worst pain imaginable’ [13]. The complete questionnaire in German language is provided in the Supplements.

### Statistical analysis

The statistical evaluation was carried out using SPSS version 21 (SPSS Inc. Chicago, IL). Statistical significance was assumed at a *p* value of < 0.05, trends at a *p* value < 0.1. The quantitative variables were evaluated descriptively (median and quartile range) and group differences between groups were assessed using the Mann–Whitney *U* test or the Student’s *T* test depending on normal distribution of the variables. Cross tables and chi-squared tests were used for categorical variables. A two-way ANOVA was additionally carried out to check for a significant interaction between the induction of labor and the gestational week. The data collected in 2015 were included in the descriptive and exploratory analyses, as well as a test for validity. To test the questionnaire for reliability, a second survey was carried out with 38 women 24–36 h and 48–72 h postpartum using a test–retest under the same conditions. A sufficient correlation was assumed for Cronbach's alpha > 0.6. The content validity (questionnaires created by experts from various disciplines for items already validated from other questionnaires with similar content) and the criterion validity were tested using plausible relationships between process and result items. Correlation analyses were carried out to check for criterion validity. The correlation coefficient Spearman Rho was used for the investigation of two ordinally distributed data, Kendall's Tau for the correlation of ordinally with binary-distributed data, and Phi for two binary items. The correlation coefficients were rated according to Cohen's effect size. A sufficiently high or medium correlation was assumed at ≥ 0.3.

## Results

### Study population

Figure 1 depicts the composition of the study population. From 1376 deliveries during the study period, the survey finally included 339 patients who are further treated as the ‘total’ study cohort (see Table 1). The age of the participants ranged from 18 to 42 years. A total of 35 operative-vaginal deliveries were observed, 27 (15.7%) in 172 primiparae and 8 (2.3%) in 167 multiparous women (*p* < 0.001). For subgroup comparisons between primiparae and multiparae all histories of operative deliveries (current and previous) were excluded. The collective of subgroups, therefore, consisted of 145 primiparae and 129 multiparae (Fig. 1; Table 1).

**Table 1:** Characterization of the study population

Items	Total *N* (%)	Primiparae	Multiparae	*p* value
*N*	339	145	129	
Age [years]	30.4 ± 4.46 Y	28.5 ± 4.42 Y	31.7 ± 3.81 Y	< 0.001
Duration of birth [hours]	5.15 [3.15;7.83] h	6.23 [3.93;8.38] h	4.08 [2.23;6.23] h	< 0.001
Induction of labor	139 (41%)	68 (46.9%)	39 (30.2%)	0.005
Prostaglandins	123 (88.5%)	48 (70.5%)	25 (64.1%)	
Balloon catheter	35 (25.2%)	17 (25.0%)	8 (20.5%)	
Others	16 (11.6%)	3 (4.5%)	6 (15.4%)	
Documented obstetric injuries	276 (81.4%)	138 (95.2%)	81 (62.8%)	< 0.001
Episiotomy	51 (15%)	24 (17.4%)	3 (3.7%)	
Perineal tear II	69 (20.4%)	35 (25.4%)	18 (22.2%)	
Perineal tear III/cervical laceration/high vaginal tear	13 (3.8%)	4 (2.9%)	2 (2.5%)	
Pain management during childbirth*	202 (59.6%)	106 (73.1%)	47 (36.4%)	< 0.001
Non opioids^1^	122/339 (36%)			
Opioids^2^	162/339 (47.8%)			
Nitrous oxide	21/339 (6.2%)			
PDA	47/339 (13.9%)			
Pudendus block	8/339 (2.4%)			
Analgesics postpartum^3^	132 (38.9%)	44 (30.3%)	48 (37.2%)	0.251
Birth weight newborn [gram]	3415 ± 513 g	3293 ± 544 g	3513 ± 495 g	0.001
Length newborn [centimeter]	52 ± 3 cm	51 ± 3 cm	52 ± 3 cm	0.045
Head circumference newborn [centimeter]	35 ± 2 cm	34 ± 2 cm	35 ± 2 cm	0.022

^1^Non-opioid analgesics: Butylscopolamine p.o./i.v./i.m./supp., Paracetamol supp.

^2^Opioids: Fentanyl i.v., Pethidine i.m., Meptazinol i.m.

^3^No. for postpartum non-opioid request/use

Mean ± standard deviation (SD), Median [25;75].

The mean age of all patients was 30.4 (± 4.5) years; 28.5 (± 4.4) years in the primiparae group and 31.7 (± 3.8) years in the multiparae group. Significant differences between the two subgroups were found with regard to age, the duration of birth, the frequency of operative-vaginal deliveries and obstetric injuries, the request for analgesics during childbirth and the size of the infant (Table 1). Overall, 132 (38.9%) patients during labor and 198 (58.4%) postpartum did not request or receive analgesic therapy. In the subgroups the respective proportions were 24.8%/65.5% (primiparae) and 62.0%/58.9% (multiparae).

**Fig. 2:**
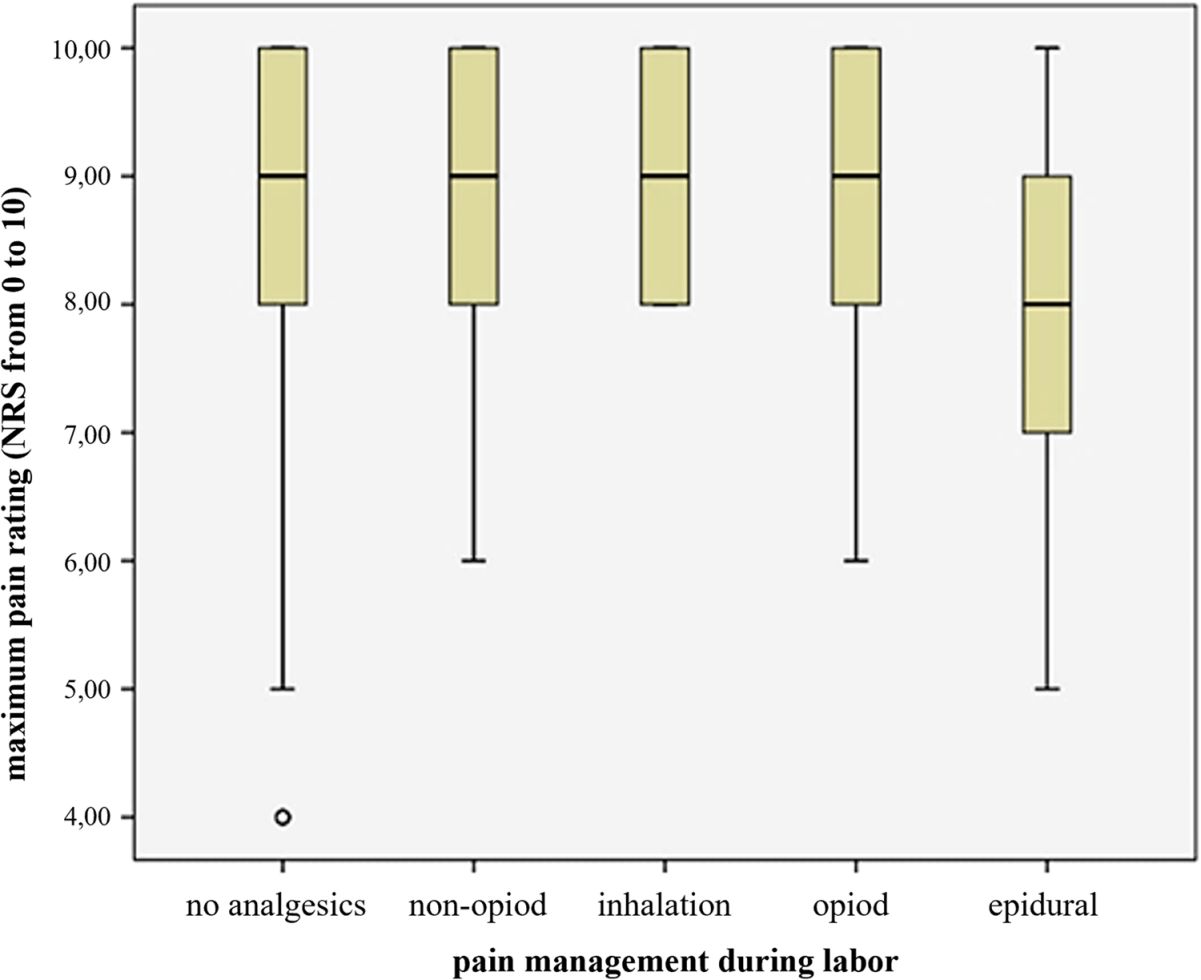
Maximum pain during labor in relation to pain management (study cohort *N* = 339).Pain levels were estimated on a numeric rating scale (NRS) from 0 (no pain) to 10 (most severe pain imaginable). The boxplots depict medians with quartile ranges

Our survey shows that giving birth is retrospectively perceived as a very painful experience (Median 9 [8–10] on the NRS) (Table 2). A median NRS of 4 [3–6] as in the postpartum period is traditionally considered as being beyond the indication threshold for systematic pain management. In general, there were no differences between primiparae and multiparae for perception of pain during labor and postpartum. 48.7% of the primiparae and 20.9% of the multiparae reported the labor pain as ‘too long’ or even ‘unbearably long’ and about one-third wished to have asked for more analgesics. In contrast, the personal involvement into decision-making and the satisfaction with the pain regimen used were high. We observed a positive relation between the severity of pain and the request for analgesics, which does not mirror in the median NRS values (Kendells Tau, total *p* = 0.035; primiparae *p* = 0.056; multiparae *p* = 0.455). The questionnaire in use did not explicitly ask about pain relief by analgesics during labor (see “Discussion”). Nevertheless, women under epidural analgesia experienced less pain during labor, than through all other analgesic administrations (*p* = 0.088; Fig. 2).

**Table 2:** Questionnaire results

Item	Total *N* = 339		Primiparae *N* = 145		Multiparae *N* = 129		*p* value
	*N*	Med [25;75]	*N*	Med [25;75]	*N*	Med [25;75]	
Maximum pain during labor	338	9 [8;10]	144	9 [9;10]	129	9 [8;10]	0.852
Duration of maximum pain	334		145		127		< 0.001
None Bearably long Too long Unbearably long	7 204 101 22	2.1% 61.1% 30.2% 6.6%	3 72 57 13	2.1% 49.7% 39.7% 9.0%	3 97 19 8	2.4% 75.2% 14.7% 6.2%	
Involvement in decision making	316	9 [7;10]	138	9 [6.75;10]	115	9 [6;10]	0.443
Satisfaction with pain managment	294	8 [5;9.25]	130	8 [5;9.25]	106	8 [5.75;9.25]	0.595
Wish to have received more analgesics	325	107 (32.9%)	141	51 (36.2%)	123	40 (32.5%)	0.604
Maximum pain postpartum	338	4 [3;6]	145	4 [2.5;5]	128	4 [3;6]	0.267
Satisfaction with postpartum pain management	289	9 [7;10]	124	9 [7;10]	106	9 [7;10]	0.892
Satisfaction with midwifery care	315	10 [9;10]	134	10 [9;10]	120	10 [9;10]	0.813

Figures are given as median (med.) and interquartiles [25;75] or percentages, were applicable (%). *N* given for each item separately: number of subjects who did answer the respective question

**Fig. 3:**
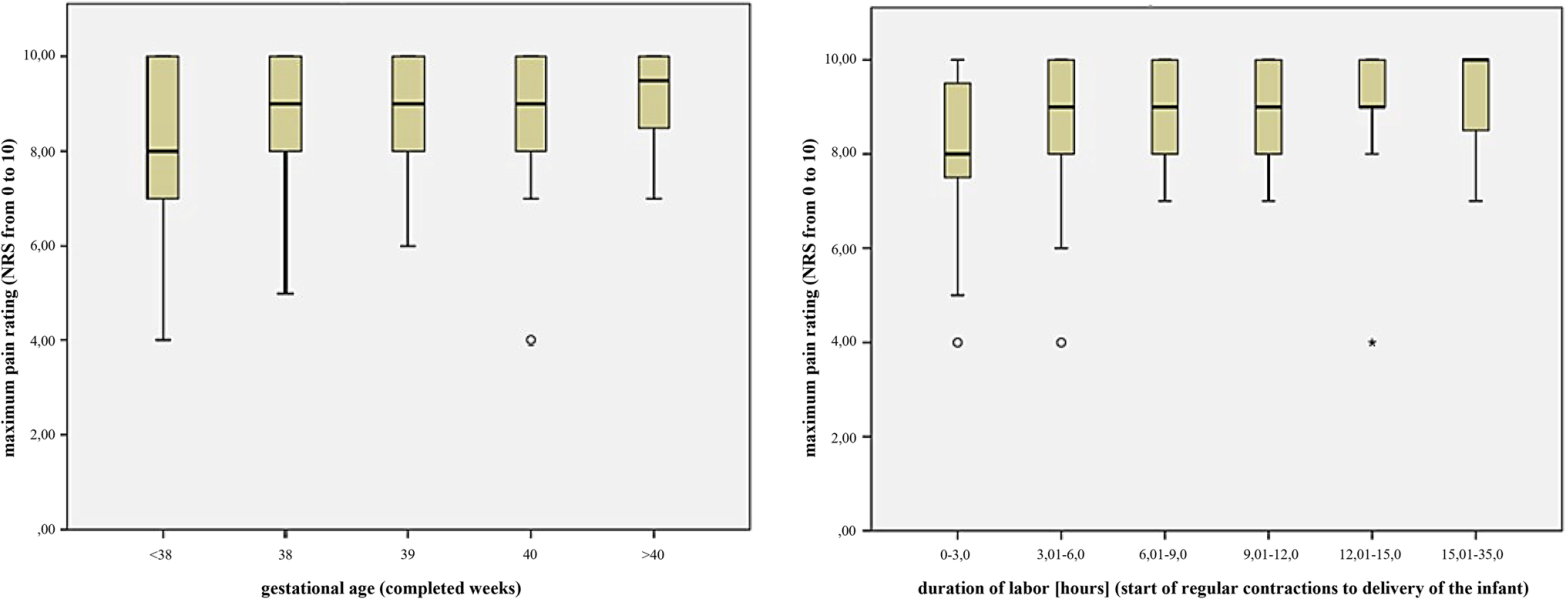
Relation between pain perception during labor, gestational age, and duration of labor (primiparae, *N* = 145). Pain levels were estimated on a numeric rating scale (NRS) from 0 (no pain) to 10 (most severe pain imaginable). The boxplots depict medians with quartile ranges

**Fig. 4:**
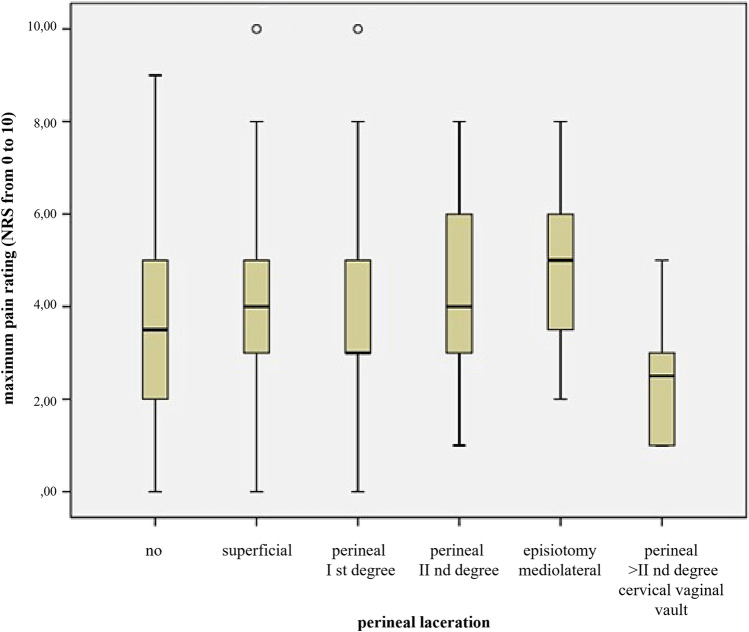
Postpartum pain experience and severity of perineal lacerations (total, *N* = 339). Pain levels were estimated on a numeric rating scale (NRS) from 0 (no pain) to 10 (most severe pain imaginable). The boxplots depict medians with quartile ranges

Table 3 and Fig. 3 give an overview on the interrelation between obstetric factors and perception of pain during labor and postpartum in primiparae who delivered spontaneously. Gestational age, duration of birth, and neonatal biometric factors are positively correlated to intrapartum pain (Table 3; illustration in Fig. 4).

**Table 3:** Labor pain and obstetric factors in primiparae (*N* = 145)

Item	Maximum pain during labor		Maximum pain postpartum	
	Correlation	*p* value	Correlation	*p* value
Maternal age	– 0.022	0.792	– 0.193	0.02
Gestational age	0.167	0.045	0.032	0.7
Duration of labor	0.197	0.018	– 0.095	0.256
Neonatal weight	0.304	< 0.001		
Neonatal length	0.325	< 0.001		
Neonatal head Circ	0.334	< 0.001		
	Median [25/75]		Median [25/75]	
Induction of labor	9 [8;10] vs. 9 [8;10]	0.075	4 [2;5] vs. 3 [2.5;5.5]	0.83
Prostaglandin vs. Balloon ripening	9 [8;10] vs. 10 [7.5;10]	0.942	4 [3;6] vs. 3 [1;4.5]	0.032

As during labor, higher pain intensity was related to an increased request for analgesic in the postpartum period (median 4 [3;6] vs. 4 [2;5], *p* = 0.014). Interestingly, higher-degree of perineal tears was associated with less pain in the postpartum period (*p* = 0.056; Fig. 4).

Pain levels were estimated on a numeric rating scale (NRS) from 0 (no pain) to 10 (most severe pain imaginable). The boxplots depict medians with quartile ranges.

Satisfaction with pain management was acceptable overall and similar between subgroups. In the correlations analysis, we observed a significant inversed correlation between satisfaction and wish for more analgesics and duration of labor. On the other hand, satisfaction with midwifery care and involvement in pain management significantly correlated with higher overall satisfaction levels (Table 4). Exemplary data in Table 4 illustrate the validation process using Cohen’s correlation. All correlations were statistically significant and showed an average strength of the correlation > 0.3 (r).

**Table 4:** Validation process

Cross correlation	Duration of labor	Involvement in pain management	Request for more analgesics	Satisfaction with midwifery care
Satisfaction with pain management	– 0.363	0.545	– 0.423	0.63

### Reliability

In the subsample of 38 subjects surveyed between July and October 2017 test–retest reliability was assessed using Cronbach’s alpha. Only 2 out of 21 items of the questionnaire were below Cronbach’s alpha < 0.6. (Question 8: Are you affected by the pain—When caring for the child? Yes / No (Cronbach's alpha 0.4), Question 18: Have you used or received non-medicinal methods for pain relief? Yes relaxation (Cronbach's alpha 0.5)). On average, correlations were > 0.9. Thus, the questionnaire achieved a high test–retest reliability and, apart from two items, proved to be reliable (figures given in the Supplements).

## Discussion

Although childbirth is known to be one of the most painful experiences, there is no validated instrument in German language designed specifically for assessing pain during or after vaginal birth. Our international data research revealed hardly any studies that examined questionnaires about pain during vaginal birth [14]. To our knowledge, this is the first prospective, non-randomized, unblinded survey on subjective pain experience during and after vaginal delivery (outcome questionnaire) and its association with demographic and obstetric aspects (process questionnaire). As a first step, and to homogenize the testing population for validation purposes, the questionnaire is kept in German. Excluding patients after operative deliveries from the subgroup analysis, we aimed to reduce confounders for the evaluation of pain, since women after instrumental birth were expected to have different levels of suffering.

Women often hesitate to request pain medication because they are afraid of possible side effects on the process of labor itself, the child’s health or the possibility of breast feeding [15, 16]. Knowledge and attitude of healthcare providers have a relevant influence on pain management during labor [17]. Nevertheless, there is a risk of reduced function, chronification of pain, and an augmentation of mental disorders in the puerperium related to pain experience [18, 19]. Therefore, a comprehensive education regarding the options of peripartum pain therapy is indispensable. On the other hand, an effective and easy-to-use tool for peripartal pain assessment based on PROs is needed to assess the quality of pain management in obstetrics. In addition to a detailed medical history, the women should be asked about fears and desires regarding labor to adapt the pain management accordingly.

Very high levels of pain intensity during birth have been described previously in the literature [1]. The high pain ratings of a median of 9 on a numeric rating scale (NRS) from 0 (no pain) to 10 (most severe pain imaginable) during childbirth and a median of 4 after childbirth in this study confirm these findings and underline the requirement to introduce a standardized pain survey policy that is specifically adapted to vaginal deliveries. According to current studies, a cut-off value of 4 on the NRS is a tolerable pain threshold for acute and postoperative pain [20]. Although these cut-off values might not apply to all types of painful procedures, a pain intensity level of 9 indicates inadequate treatment. The overall satisfaction with intrapartum care, though, remains contradictory and may indicate that these standards are not directly applicable particularly to labor pain. The problem of ceiling effects of birth pain has to be considered as well. Differentiation in the upper range is difficult due to the very high maximum pain values, especially during childbirth.

The personal obstetric history showed no influence on the maximum pain levels recorded in this study. However, the analysis did not examine the number of vaginal deliveries in relation to the maximum pain. It is, therefore, not possible to say whether a certain number of vaginal deliveries are associated with reduced pain severity. The gestational age, as well as the infant's dimensions, had the expected significant influence on the maximum pain in the group of primiparae. It is surprising that this effect could not be demonstrated in the case of multiparae compared to primiparae, although the children of the multiparae were significantly larger and heavier in comparison. The infant’s biometrics should, therefore, be included in future studies on pain management.

Women with balloon induction reported significantly less pain compared to women after prostaglandin induction. As a limitation, it must be mentioned that the analysis was carried out with a small number of cases. In addition, the prostaglandins were not further classified according to the type of prostaglandin, dosage or the way of application. Currently, it is questionable why the method of induction may have an impact on pain intensity after birth and not during birth.

Regional analgesia such as epidural (synonymously used peridural analgesia – PDA) tended to subjectively reduce the pain felt during childbirth. This result reflects the superiority of regional analgesia, which has already been proven in several randomized studies, especially in comparison to opioid therapy [9, 21]. Nevertheless, the questionnaire failed to differentiate whether pharmacological interventions other than PDA resulted in pain relief since there was no specific prior-posterior question addressing this issue. Therefore, one must not jump to the premature conclusion that interventions other than PDA may be of no use in labor pain management.

With a median of 5 for maximum postpartum pain, women with an episiotomy reported significant higher pain scores compared to all other perineal or vaginal traumas. This correlates with previous studies, which also showed that patients who had an episiotomy indicated more pain after the episiotomy, as well as in the puerperium, compared to a second degree perineal tear [22, 23]. Women with higher-grade injuries reported less pain in the postpartum period. This seemingly contradictory result is likely to be associated to the circumstance that these patients were preferably treated under general or epidural/spinal analgesia and preventive postoperative pain management was applied. It underlines the importance of a systematic approach to postpartum pain relief. Special attention with regard to effective pain therapy should, therefore, be given to patients after an episiotomy. Another group requiring more attention are particularly young primiparae as they represent a risk group for increased postoperative pain [24], even after vaginal delivery.

With the aim of improving quality in postoperative pain therapy, the QUIPS project has been established for many years and is used in various disciplines. Several studies showed that the questionnaire is a useful tool for quality improvement and can be used in routine clinical practice [25–27]. Multicenter use of this tool allows benchmarking and opens the possibility to identify best clinical practice and learn from each other, resulting in better care for patients. Therefore, based on the experience earned in the application of the QUIPS questionnaire after cesarean section, this study group developed a version for vaginal delivery.

The questionnaire uses NRS to assess maximum pain and satisfaction with the care provided by the midwife and pain therapy. In comparison to other one-dimensional scales, the NRS shows the best results in terms of sensitivity, error rate, handling, and acceptance[28, 29]. The questionnaire not only records the impairment in mood, but also differentiates between the individual feelings (e.g., sadness, fear, excessive demands, etc.). Overall, the questionnaire is a versatile, easy-to-use and a well-accepted survey tool for interviewers and patients (response rate 80.1%), that can easily be integrated into everyday clinical practice[25–27].

### Quality criteria of the questionnaire

The test–retest reliability always depends on the time interval between the two measurements. Because measurement was only possible at a short interval, there is a risk of memory effects and an artificially increased test–retest reliability[30]. The difficulty in pain assessment is that pain and memory of pain is a dynamic trait. The relevant correlation of 0.6 was missed only for the item “difficulties with caring for the child” due to pain after birth. The information changed exclusively from "impairment" to "no impairment". This may be explained by the fact that after a time interval of more than 48 h, the patients were more experienced in dealing with the newborn. The improved care of the child through reduced pain in the course is also possible. All other assessed items showed robust reliability. The lack of a suitable external criterion of quality hindered the procedure of external validation to be carried out. At the moment, no questionnaire to determine construct validity is available. This only left the verification of content and criterion validity. The questionnaire was created by experts from various disciplines based on an already validated questionnaire that has been established for years for postoperative pain [31]. Care was taken to ensure that the questionnaire has a multidimensional approach in describing the quality of the results. In addition to maximum pain, functional effects of pain, psychological aspects, and patient satisfaction were taken into account. This multidimensional approach coincides with the demands made by other experts to describe the quality of pain therapy [32–35]. The selection of the measurement method for satisfaction and maximum pain, using NRS, has already been validated in other studies [28, 29]. Overall, it can be assumed that the questionnaire covers all facets of pain and the associated restrictions and thus has good content validity.

The criterion validity was checked against theoretical criteria, which should demonstrate the plausibility of the results. The correlation analyses carried out show that the results are conclusive and as expected. In validation studies about chronic pain, weak to medium correlations were reported with regard to an external criterion[36–39]. The other significant relationships further support the plausibility of the results. In summary, it can be concluded that the questionnaire allows statements about maximum pain and its associated restrictions with sufficient reliability.

Another possibility would be the use of external criteria to assess pain intensity or functional restrictions, e.g., in the form of medical staff notes. Furthermore, the pain-related functional restrictions could be verified with a variety of activities. In the best case, several validity criteria are used for each item to identify the best possible external criterion.

### Limitations

Studies have shown that the pain memory is not exact and strongly context-dependent [29]. The results can also be influenced by a memory distortion effect called “recall bias” [40, 41]. The questionnaire on pain during childbirth was carried out retrospectively, the questionnaire on pain after childbirth in turn relate to the current condition and is, therefore, not, or less affected by memory effects. Pain intensity and pain memory after trauma or after surgery decrease over time. This effect has also been observed for labor pain[42]. Due to the design of the survey, no statement can be made about the further development or duration of the pain after birth. It cannot be ruled out, but is unlikely to be a major setback, that the patients were exposed to a peak of pain at the time of the interview. The risk of divergent answers was minimized by the independent responses of the patients and a survey by personnel not involved in woman’s care [43]. An influence of the “Hawthorne effect” or the presence of relatives on the indication of pain intensity cannot be ruled out with certainty [44, 45]. The study has so far been carried out monocentrically at only one German hospital (Jena University Hospital) and is, therefore, not a representative cross-section of German hospitals. Additionally, it has so far only been possible to interview German-speaking patients. Therefore, there are some limits to the generalizability, especially regarding the influence of the cultural background in dealing with pain [46]. However, it is planned to provide versions in several other languages, as it was done in PAIN OUT, the international part of the QUIPS project.

## Conclusion

Giving birth is a severely painful process; a phenomenon requiring more attention of care-givers. Even when reporting overall satisfaction with their situation, a relevant number of women, if asked, report significant subjective pain levels on NRS that would trigger medical pain relief in a postoperative ward. The reported pain levels indicate inadequate pain management. Therefore, systematic assessment of pain perception in the maternity ward is a pending issue. We present a questionnaire of acceptable validity specifically designed to direct pain management sub partu and postpartum. It may be useful to assess department-specific changes of standards of procedure and propel standardized comparison between departments as a means for applied clinical science. In result to this validation study, we incorporated a question specifically asking the effect of pharmacologic intervention on labor pain perception. To additionally address women of a different cultural background, which do not have sufficient German language skills, the validated version of this questionnaire will be translated and may serve as an instrument to compare cultural differences of labor pain perception as well. Even severe pain sub partu does not necessarily lead to dissatisfaction with intrapartum care.

## Supplementary Information

Below is the link to the electronic supplementary material.

Supplementary file1 (PDF 445 KB)

## Data Availability

The raw/processed data required to reproduce these findings cannot be shared at this time due to legal or ethical reasons. The questionnaire will be available on the QUIPS platform at http://www.quips-projekt.de/.
