# Supplementary material for: Peripartal pain perception and pain therapy: introduction and validation of a questionnaire as a quality instrument
Source: Arch Gynecol Obstet. 2021 Sep 20;305(6):1409–19. doi: 10.1007/s00404-021-06246-w (PMC9166832; doi:10.1007/s00404-021-06246-w)
Supplement: Supplementary file 1 — Supplementary file1 (PDF 445 KB) [file 404_2021_6246_MOESM1_ESM.pdf]

Supplements

Supplement 1:
Outcome questionnaire

QUIPS Ergebnis-Fragebogen

Sehr geehrte Patientin,

Bei den folgenden Fragen geht es um Ihre **Schmerzen** <u>WÄHREND Ihrer Entbindung</u> **(darunter ist die Zeit vom Einsetzen der ersten Wehen bis zur Geburt gemeint):**

1. Wie stark waren Ihre Schmerzen WÄHREND der Entbindung?
Kreuzen Sie bitte die Zahl auf der Skala an, die für Ihren Maximalschmerz während der Entbindung zutrifft („0“ bedeutet Schmerzfreiheit und „10“ stärkster vorstellbarer Schmerz).

0	1	2	3	4	5	6	7	8	9	10

Kein Schmerz — Stärkster vorstellbarer Schmerz

2. Nach Ihrer eigenen Einschätzung, litten Sie unter starken Schmerzen:
Mit starken Schmerzen sind Schmerzen von 6 oder mehr in der o.g. Skala gemeint

❑ Unerträglich lange ❑ Zu lange ❑ Erträglich lange ❑ Gar nicht

3. Zu welchem Zeitpunkt der Entbindung waren die Schmerzen am stärksten?
- ❑ Beim Einsetzen der ersten Wehen
- ❑ Während der Untersuchungen durch Arzt oder Hebammen
- ❑ Während der Wehen
- ❑ Als ich pressen musste
- ❑ Als das Kind geboren wurde
- ❑ Während der Entbindung des Mutterkuchens
- ❑ Als ich genäht wurde
- ❑ Nach der Entbindung
- ❑ Sonstiges: ________________

4. Wurden Sie in dem von Ihnen gewünschten Maß zu **Entscheidungen** bezüglich **Schmerztherapie beteiligt?**

0	1	2	3	4	5	6	7	8	9	10

Gar nicht — Völlig ausreichend

5. Bitte kreuzen Sie an, wie **zufrieden** Sie mit dem Ergebnis Ihrer **Schmerztherapie WÄHREND** der Entbindung sind:

0	1	2	3	4	5	6	7	8	9	10

Völlig unzufrieden — Sehr zufrieden

6. Hätten Sie sich WÄHREND der Entbindung mehr Schmerzmittel gewünscht, als Sie erhalten haben? ❑ Ja ❑ Nein

Folgende Fragen beziehen sich auf Ihre <u>**Schmerzen NACH der Entbindung:**</u>

7. Wie stark waren Ihre stärksten Schmerzen NACH der Entbindung?

0	1	2	3	4	5	6	7	8	9	10

Kein Schmerz — Stärkster vorstellbarer Schmerz

Copyright Winfried Meißner/ Uniklinikum Jena. Nutzung durch Dritte vorbehaltlich Genehmigung durch DGAI/ DGCH Version 3.2

Die nächsten Fragen beziehen sich darauf, ob bestimmte Tätigkeiten seit der Entbindung **durch den Schmerz beeinträchtigt** sind.
Mit „beeinträchtigt" ist gemeint, dass die Tätigkeit unmöglich oder nur unter großer Mühe möglich ist.

8. Sind Sie **durch die Schmerzen** beeinträchtigt:
- **a.** beim **Gehen?** ❑ Ja ❑ Nein
- **b.** beim **Sitzen?** ❑ Ja ❑ Nein
- **c.** bei der **Versorgung des Kindes?** ❑ Ja ❑ Nein
- **d.** beim **Stillen?** ❑ Ja ❑ Nein
- **e.** beim **Schlafen?** ❑ Ja ❑ Nein

9. Wie schwer fallen Ihnen **wegen Schmerzen** folgende Aktivitäten seit der Entbindung?

	**problemlos**	**wenig**	**mäßig**	**stark**	**unmöglich**
Gehen	❑	❑	❑	❑	❑
Sitzen	❑	❑	❑	❑	❑
Kind versorgen	❑	❑	❑	❑	❑
Wasserlassen	❑	❑	❑	❑	❑
Stillen	❑	❑	❑	❑	❑
Schlafen	❑	❑	❑	❑	❑
Husten/Luft holen	❑	❑	❑	❑	❑
Stuhlgang	❑	❑	❑	❑	❑

10. Wo haben Sie jetzt Schmerzen? (Mehrfachnennungen möglich)
- ❑ Keine Schmerzen
- ❑ Oberbauch
- ❑ Unterleib
- ❑ Scheide
- ❑ Kopf
- ❑ Naht
- ❑ Damm
- ❑ Steiß
- ❑ Schambein
- ❑ Rücken
- ❑ Hämorrhoiden/After

11. Sind Sie seit der Entbindung in Ihrer Stimmung beeinträchtigt?
❑ Ja ❑ Nein

Falls ja, wie stark?

0	1	2	3	4	5	6	7	8	9	10

Gar nicht — Völlig beeinträchtigt

Welche Empfindungen treffen auf Sie zu:
- ❑ Traurigkeit
- ❑ Überforderung
- ❑ Enttäuschung
- ❑ Aggression
- ❑ Gleichgültigkeit
- ❑ Sonstiges:__________________
- ❑ Angst (z.B. davor, ihr Kind nicht gut genug versorgen zu können)
- ❑ Gefühl, keine Beziehung zu Ihrem Kind aufbauen zu können

12. Hätten Sie sich NACH der Entbindung mehr Schmerzmittel gewünscht als Sie erhalten haben? ❑ Ja ❑ Nein

13. Bitte kreuzen Sie an, wie **zufrieden** Sie mit dem Ergebnis Ihrer **Schmerztherapie NACH** Ihrer Entbindung sind

0	1	2	3	4	5	6	7	8	9	10

Völlig unzufrieden — Sehr zufrieden

Copyright Winfried Meißner/ Uniklinikum Jena. Nutzung durch Dritte vorbehaltlich Genehmigung durch DGAI/ DGCH Version 3.2

Die Fragen auf dieser Seite beziehen sich allgemein auf Ihre Meinung über die Schmerztherapie während der Entbindung und Entbindungsvorbereitenden Maßnahmen

14. Was haben Sie vor der Entbindung über Schmerzmittel während der Entbindung gedacht?
❑ Ich wollte mir keine Schmerzmittel geben lassen
❑ Ich wollte selbst entscheiden, ob ich ggf. Schmerzmittel brauche
❑ Ich wollte die Entscheidung dem Arzt bzw. der Hebamme überlassen
❑ Ich wollte auf jeden Fall Schmerzmittel gegen Schmerzen

15. Hat sich Ihre Meinung dazu geändert? ❑ **Nein** ❑ **Ja**

16. Welche der folgenden Maßnahmen haben Sie zur Entbindungsvorbereitung VOR der Entbindung angewendet:

❑ keine
❑ Entbindungsvorbereitungskurs
❑ Akupunktur
❑ Dammmassagen
❑ Bauchmassagen
❑ Tees
❑ Meditation
❑ Sitzbäder
❑ Homöopathie
❑ Schwangerschaftsgymnastik
❑ Hypnose
❑ Leinsamen

17. Wurden Sie über die verschiedenen **Möglichkeiten der Schmerztherapie** informiert? ❑ Ja ❑ Nein

18. Haben Sie **nicht-medikamentöse** Methoden zur **Schmerzlinderung** benutzt oder erhalten?
❑ Ja ❑ Nein

Falls ja, welche (Mehrfachnennung möglich)?

❑ Kühlkompressen
❑ Akupunktur
❑ Homöopathie
❑ Positionswechsel
❑ Heißes Bad
❑ Aromatherapie
❑ Andere__________
❑ Wärme
❑ Beten
❑ Gespräche
❑ Meditation
❑ Umhergehen
❑ Entspannung
❑ Tiefes Atmen
❑ Massagen
❑ TENS (Transkutane **elektrische** Nervenstimulation)

19. Waren Sie während der Schwangerschaft in Behandlung wegen ständigen Schmerzen, die **drei Monate oder länger** andauerten?
❑ Ja ❑ Nein

Vom Befragenden auszufüllen: Patientin wurde interviewt: ❑ Ja ❑ Nein

Copyright Winfried Meißner/ Uniklinikum Jena. Nutzung durch Dritte vorbehaltlich Genehmigung durch DGAI/ DGCH Version 3.2

FT 1 Bitte kreuzen Sie an, wie **zufrieden** Sie mit der **Hebammen**betreuung **WÄHREND** Ihrer Entbindung sind:

0	1	2	3	4	5	6	7	8	9	10

Völlig unzufrieden — Sehr zufrieden

FT 2 Wie **umfangreich** schätzen sie die Betreuung durch die **Hebamme** während der Entbindung ein?

0	1	2	3	4	5	6	7	8	9	10

Gar nicht — Völlig ausreichend

Copyright Winfried Meißner/ Uniklinikum Jena. Nutzung durch Dritte vorbehaltlich Genehmigung durch DGAI/ DGCH Version 3.2

Supplement 2:
Test-retest reliability using Cronbach's alpha shown for all questions of the result questionnaire. Only 2 out of 21 items of the questionnaire were below Cronbach's alpha <0.6.

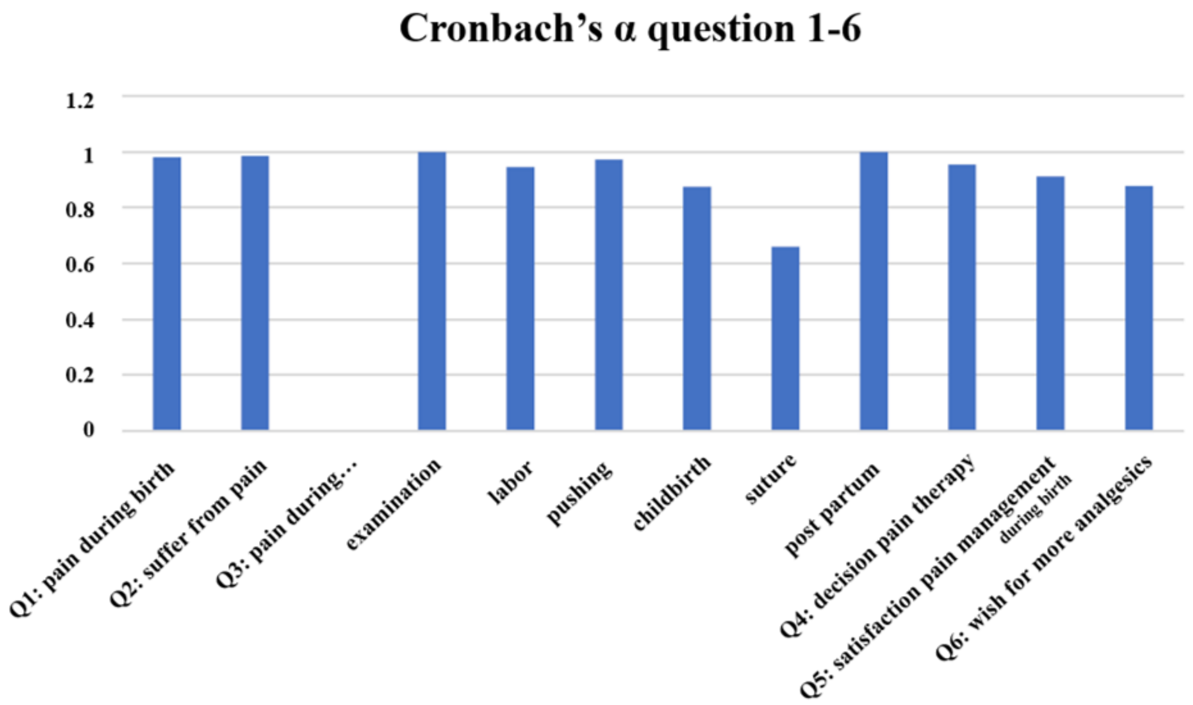

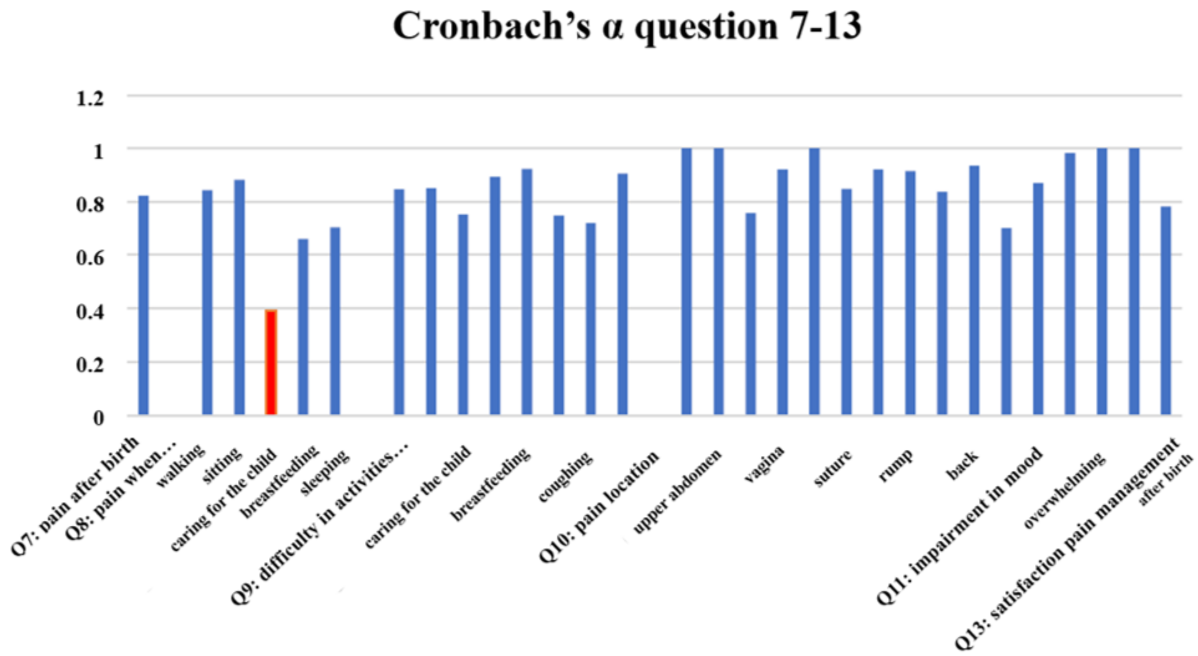

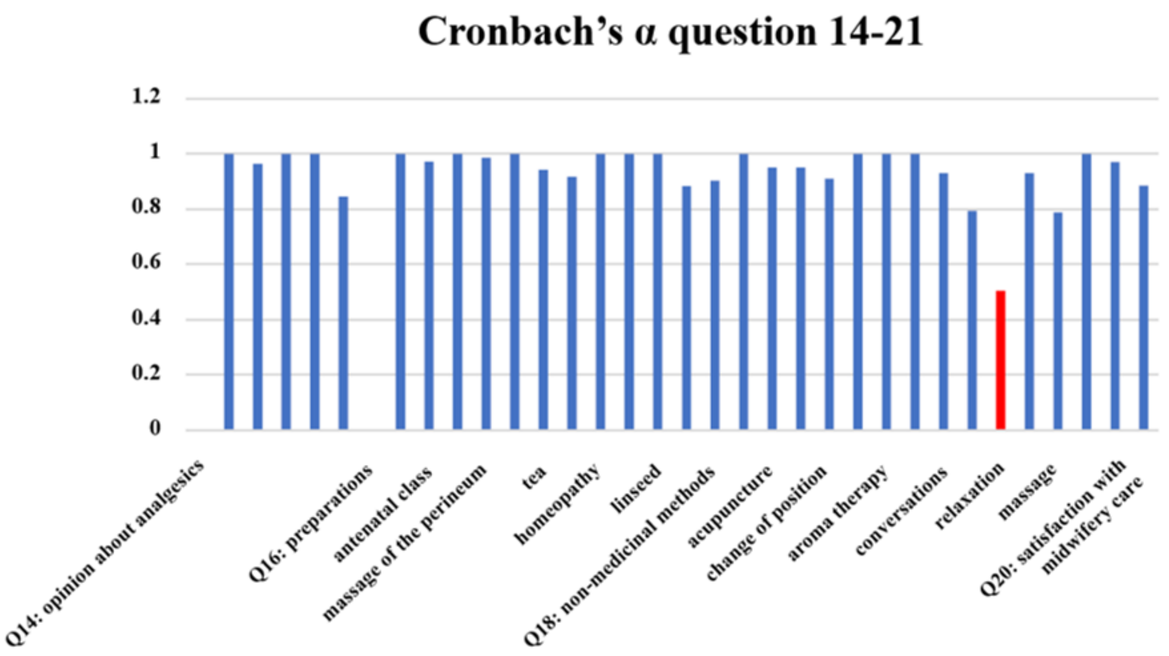
Cronbach's α question 14-21
1.2
1
0.8
0.6
0.4
0.2
0
Q14: opinion about analgesics
Q16: preparations
antenatal class
massage of the perineum
tea
homeopathy
linseed
Q18: non-medicinal methods
acupuncture
change of position
aroma therapy
conversations
relaxation
massage
Q20: satisfaction with midwifery care
